# The Impact of Destructive Climatic Factors on the Mechanical and Performance Properties of Structural Materials

**DOI:** 10.3390/ma18132970

**Published:** 2025-06-23

**Authors:** Paweł Widomski, Przemysław Maksymowicz, Oliwia Trzaska, Paulina Mayer-Trzaskowska, Paweł Kaczyński, Anna Berbesz-Wyrodek, Barbara Gronostajska, Waldemar Bober, Michał Kogut

**Affiliations:** 1 Center for Materials Engineering and Metal Forming, Wrocław University of Science and Technology, Lukasiewicza 5, 50-371 Wrocław, Poland; 2 Department of Ecology, Biogeochemistry and Environmental Protection, University of Wrocław, 50-137 Wrocław, Poland; 3 Department of Lightweight Elements Engineering, Foundry and Automation, Faculty of Mechanical Engineering, Wrocław University of Science and Technology, Łukasiewicza 7, 50-371 Wrocław, Poland; 4 Faculty of Architecture, Wrocław University of Science and Technology, Bolesława Prusa 53/55, 50-317 Wrocław, Poland; anna.berbesz@pwr.edu.pl (A.B.-W.);; 5 Metaloplastyka Marian Ostrowski, 55-330 Błonie, Poland

**Keywords:** destructive factors, structural materials, artificial aging, innovative materials in architecture, resilient architectural materials

## Abstract

This study investigates the effects of destructive climatic factors on the mechanical and performance properties of various structural materials, encompassing both polymers and metals. Over recent decades, the growing adoption of synthetic polymers has revolutionized engineering applications, yet their susceptibility to environmental degradation poses significant challenges. This research emphasizes the need for comprehensive testing under both operational and environmental stressors, including extreme temperatures, UV radiation, and moisture, to assess material durability and performance. Mechanical tests were conducted at ambient (25 °C) and low temperatures (−50 °C) to evaluate the strength and strain responses of selected materials. Additionally, a 12-month accelerated aging process using UV radiation and elevated temperatures was performed to simulate long-term environmental exposure. Parameters such as Shore D hardness, gloss, and mass were measured at regular intervals to quantify material degradation. The results revealed significant differences in performance across material types. Among polymers, laser-extruded and milky plexiglass, as well as solid polycarbonate, exhibited satisfactory resistance to aging, with minimal changes in mechanical properties. However, high-impact polystyrene displayed substantial deformation and hardness loss after prolonged UV exposure. For metals, aluminum and stainless steel (304 and 316) demonstrated exceptional durability, retaining structural and aesthetic properties after 12 months of accelerated aging, whereas galvanized steel exhibited pronounced corrosion. The study highlights the critical interplay between mechanical loading and environmental factors, stressing the importance of material selection tailored to specific climatic conditions. It further underscores the value of integrating experimental findings with predictive models, such as finite element analysis, to enhance the design and longevity of engineering materials. The findings provide actionable insights for industries operating in temperate climates, where materials are subjected to diverse and cyclic environmental stressors. Recommendations are offered for selecting resilient materials suitable for protective housings and structural components.

## 1. Introduction

In recent decades, the realm of materials science has witnessed a proliferation of new materials, each finding diverse applications in engineering and technology. Notably, the ascendancy of synthetic polymers has reshaped industrial landscapes, progressively displacing conventional metallic alloys. As early as the 1990s, plastics surpassed steel in production volume, and since then, their utilization has surpassed expectations across various sectors [1]. Synthetic polymers, owing to their versatility, have become ubiquitous in everyday objects, serving as enclosures and structural components in a myriad of devices. However, their susceptibility to environmental degradation and limited durability remain significant challenges. While plastics are commonly employed for single-use or limited reusability packaging, their application in critical machinery components or structures exposed to harsh environmental conditions such as sunlight and water remains less frequent [2].

It’s imperative to note that synthetic polymers exhibit diverse properties, with some being well-suited for such applications while others succumb to environmental stressors within mere hours of exposure to sunlight. The myriad of additives and varying material qualities further complicate straightforward assessments of a material’s suitability for challenging environments. Hence, there arises a need for comprehensive testing methodologies to evaluate material durability concerning specific degrading factors. These factors encompass both operational conditions, including static and dynamic mechanical loads, and environmental variables such as climatic and atmospheric influences. The impact of static, cyclic, and dynamic mechanical loads can vary significantly, necessitating meticulous consideration of both externally applied forces and spontaneous disturbances induced by wind or operational misuse. Material property profiles derived from strength tests conducted at various strain rates can serve as invaluable inputs for structural design calculations, aiding in material and cross-sectional profile selection in engineering applications [3].

The influence of climatic factors is regionally specific, with distinct climatic zones dictating the prevailing environmental conditions. Typically, five climatic zones are recognized: equatorial, tropical, subtropical, temperate (warm and cool), and polar. Poland, along with much of Europe and North America, falls within the temperate climate zone, characterized by average monthly temperatures ranging from −30 °C to 20 °C. The subtropical climate zone, covering the remainder of Europe, experiences average temperatures ranging from 5 °C to 35 °C. Broadly speaking, temperatures in these zones seldom exceed −50 °C to +50 °C, with precipitation, including rain and snow, playing a significant role in shaping environmental conditions [4]. The combined effects of precipitation and solar radiation can substantially impact material durability, particularly in the case of synthetic polymers [5].

Existing literature underscores the imperative of conducting comprehensive investigations given the intricate interplay between mechanical loading and climatic factors, which collectively influence material longevity. Recent studies have delved into various aspects of material durability in moderate environmental conditions, shedding light on methodologies for assessing and enhancing the resilience of engineering materials. These studies have explored various degradation pathways of materials, including physical, chemical, and biological processes, highlighting the complex interactions with the surrounding environment and their impact on the materials’ long-term performance. For example, exposure to temperature fluctuations, UV radiation, a factor contributing to photo-oxidation, and humidity can accelerate the physical degradation of polymers, leading to cracking, embrittlement, and fragmentation [6]. Recent advancements in UV-curable polymers, such as the development of photosensitive resins with exceptional resistance to UV radiation and thermal stability up to 405 °C [7], highlight the importance of designing materials capable of withstanding prolonged UV exposure. These findings provide valuable insights into the mechanisms of polymer degradation and potential strategies for enhancing their durability under environmental stressors.

Recently, many research groups have undertaken diversified environmental impact analyses, often guided by detailed IEC standardized tests, which are conducted to assess the durability of various industrial products used in different sectors. The study by [8] investigated the relationship between climate, degradation of polymeric materials, and the degradation of photovoltaic (PV) modules, providing an overview of climate-induced PV failure modes and discussing new climate-based designs and material choices for PV modules. Ref. [9] investigated the impact of temperature and moisture on the degradation of 3D-printed polymethyl methacrylate dental materials using molecular dynamics simulations, highlighting the material’s sensitivity to pressure-induced factors and the importance of considering these conditions in design and material selection for dental applications.

Due to production constraints and the rapid advancement of research, specialized equipment has been developed to accelerate the analysis of environmental degradation processes through controlled simulations. In the search for reinforcing substances, several studies have explored the effect of stabilizers and dyes on the photostability of nylon 66 [10], as well as the influence of stabilizers and pigments on polypropylene photodegradation, showing that TiO_2_ pigment offers superior protection [11]. As an example of research using such equipment, Ref. [12] examined the impact of UV radiation and temperature on the molecular structure of polyamides (PA6 and PA66) and polypropylene (PP) under accelerated weathering conditions. Photo-oxidation effects were analyzed using FTIR spectroscopy and DSC, revealing significant changes in the hydroxyl, carbonyl, and crystalline regions of the polyamides.

Similarly, Ref. [13] studied the aging resistance of polyoxymethylene (POM), a material commonly used in the automotive industry, known for its high performance but also its susceptibility to environmental degradation. The study was conducted under simulated temperate climate conditions focused on enhancing POM’s structural integrity through nanoreinforcement and stabilizing agents. Factors such as solar radiation, water, heat, and internal stresses were considered, with mechanical properties (strength, stiffness and elongation), functional characteristics (color and gloss), and structural changes assessed using DSC and FT-IR spectroscopy.

In [14], the authors analyzed the thermal degradation mechanisms of polymeric materials, highlighting the effects of high temperatures on the decomposition of polymers and the techniques used to characterize these processes, including thermogravimetry (TG) and differential scanning calorimetry (DSC), while also addressing the impact of the polymer structure and additives on thermal stability in industrial applications. Other scientists discussed the degradation of specific polymers such as chlorosulfonated polyethylene, demonstrating how combined thermal and radiative stress can influence material performance, leading to changes in physical properties such as elongation and tensile strength [15].

Additive manufacturing, particularly Fused Deposition Modeling (FDM), enables layer-by-layer fabrication of components from thermoplastics. Ref. [16] demonstrated that accelerated UV-B aging significantly reduces the tensile and compressive strengths of 3D-printed PLA and PETG parts, highlighting their vulnerability to photodegradation and the need for careful material selection in outdoor applications. Another interesting paper [17] discussed the significant improvements in corrosion resistance achieved through nanocomposite coatings, highlighting various types such as metal-metal, metal-oxide, metal-polymer, and ceramic-metal matrices, which are effective due to electrochemical deposition methods. Similarly, Ref. [18] emphasized the enhancement of polymeric coatings for anticorrosion applications through the incorporation of nanomaterials, improving their barrier properties and addressing issues like porosity and mechanical damage.

The large number of polymeric materials and their composites has led to an increased focus on developing applied mathematical methods that could streamline costly and time-consuming screening procedures. Other studies use analytical methods like regression analysis or the Arrhenius equation to assess both natural and accelerated aging processes, as well as predict changes in mechanical properties [19,20]. Moreover, evolutionary models are used to track material degradation and changes in load-bearing capacity during aging [21]. In this context, publication [22] presented approaches to modeling the environmental aging of polymers and polymer composites, focusing on modular and multiscale methods. These models provide a systematic framework for predicting material degradation, incorporating processes such as diffusion and using accelerated testing to simulate long-term environmental exposure. 

However, recent trends show a shift toward integrated approaches, such as finite element analysis (FEA), which combine multiple aspects of material degradation into a single, comprehensive model. FEA models are increasingly used to simulate the degradation of fiber-reinforced polymer composites under environmental stresses, considering factors such as fatigue, aging, and temperature cycles, to predict long-term material behavior [23,24]. This approach is gaining importance as it provides a more multifaceted understanding of how different degradation mechanisms interact over time, improving the accuracy of lifetime predictions. 

Despite their growing use, FEA models still face challenges in fully capturing the complexity of material behavior, particularly when dealing with heterogeneous materials or all potential degradation mechanisms; therefore, these models are often combined with experimental, computational, and field studies, emphasizing a multidisciplinary approach that is essential for accurately assessing material durability and for informing the design and selection of materials for optimal performance in various environmental conditions.

Environmental factors significantly influence the durability and performance of the metals mentioned in the article, with varying effects depending on the material and exposure conditions. Aluminum, for instance, forms a protective oxide layer that resists corrosion in many environments, but in marine settings with high chloride concentrations, it is prone to pitting corrosion due to chloride ions penetrating oxide layers [25]. DC01 steel (1.0330), a carbon steel, is highly susceptible to electrochemical corrosion in humid or industrial atmospheres, as demonstrated by studies showing a 30% increase in corrosion current density under cathodic polarization in NaCl environments [26]. In contrast, stainless steel 316, enriched with molybdenum, demonstrates superior resistance to chloride-induced corrosion, as evidenced by its ability to withstand chloride concentrations below 60 °C without stress corrosion cracking [27]. Copper and its alloys, such as brass, develop patina layers that offer some protection but are vulnerable to accelerated degradation in acidic rain conditions, with studies showing 40% mechanical property loss in simulated urban acid rain [28]. Galvanized steel benefits from sacrificial zinc coatings that corrode preferentially; however, these coatings degrade rapidly in sulfur dioxide-rich industrial areas, where SO_2_ reduces zinc’s atmospheric corrosion resistance by up to 80% [29]. CuNi12Zn24 (nickel silver) resists tarnishing under moderate humidity but reacts with sulfur compounds in urban atmospheres, forming sulfides that compromise surface integrity. Stainless steel 304, while resistant to oxidation, can suffer stress corrosion cracking in high-temperature chloride environments, as shown by accelerated degradation at 816 °C in cyclic loading tests [30]. Studies have shown that temperature fluctuations and precipitation cycles exacerbate the degradation of metals like DC01 steel, reducing their thickness and mechanical integrity over time. Additionally, UV radiation combined with moisture accelerates the breakdown of protective coatings on metals like aluminum and galvanized steel, with UV-B wavelengths increasing pitting corrosion rates by 50% in marine atmospheres [31]. These interactions highlight the importance of selecting appropriate materials and protective measures tailored to specific environmental conditions for optimal long-term performance.

Material research represents one of the critical phases of a project focused on developing a multifunctional device for antiviral disinfection and health monitoring in public spaces. One of the key research stages involved the design of a modular device casing, which needed to meet specific mechanical performance requirements. The final selection of casing materials was based on multiple criteria, including moisture resistance at an IP55 protection level and resistance to mild acids commonly found in outdoor environments. For laboratory testing, 18 material samples were selected and subjected to mechanical evaluation, including colorless cast PMMA plexiglass (gs) laser, laser extruded plexiglass, OPAL milky laser plexiglass, PMMA black laser plexiglass, milled solid polycarbonate, high-impact polystyrene (HIPS) black, DIBOND composite plate (2xAl 0.21 mm PE core), engraving laminate, KRION mineral composite milling cutter, CuNi12Zn24 (nickel silver/alpaca), aluminum, DC01 steel (1.0330), stainless steel 316, copper, brass, stainless steel 304, and galvanized steel. The external casing of the device required materials with corrosion resistance and antiviral properties. From a quality assurance perspective, the casing had to fulfill three primary functions: protective, ensuring durability and resistance to environmental factors; antiviral, minimizing the presence and proliferation of viruses and bacteria; aesthetic, maintaining an attractive visual appearance. The market offers a limited number of coatings that effectively reduce viral and bacterial growth on surfaces. A particularly crucial aspect was the selection of materials in direct contact with users’ hands. To optimize material costs and the casing design, it was essential to develop hybrid material combinations through appropriate analysis of material properties, strength, and surface resistance. These considerations were fundamental in achieving an effective, cost-efficient, and functional solution for the device’s casing.

## 2. Materials and Methods

### 2.1. Mechanical Strength Testing of Materials

Mechanical tests were conducted using a Tinius Olsen H25KT strength testing machine (Tinius Olsen, Redhill, UK) according to PN-EN ISO 527:2020 and PN-EN 6892 standards. For testing at −50 °C, a chamber and Solo 2 Tinius Olsen controller were utilized. In the case of tests conducted in the chamber at −50 °C, the samples were conditioned for 5 or 10 min each time after reaching the desired temperature. The temperature measurement accuracy was ±1 °C. Table 1 summarizes the conditions under which tensile strength tests were conducted.

### 2.2. Aging Tests over a 12-Month Period Using a Xenon Lamp in a UV Chamber

**Figure 1:**
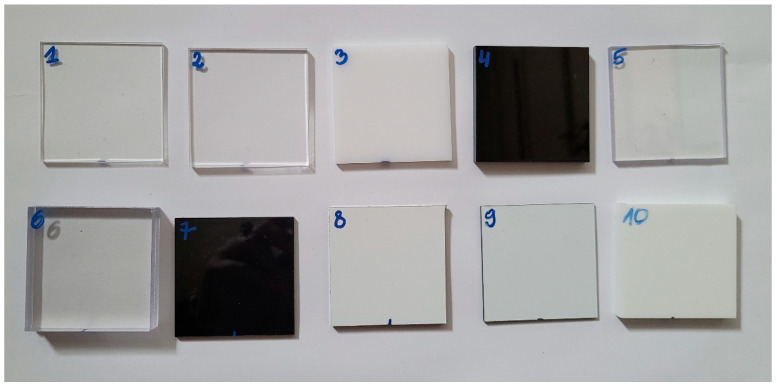
Samples for aging tests—plastic samples only, numbered as described in Table 1.

**Figure 2:**
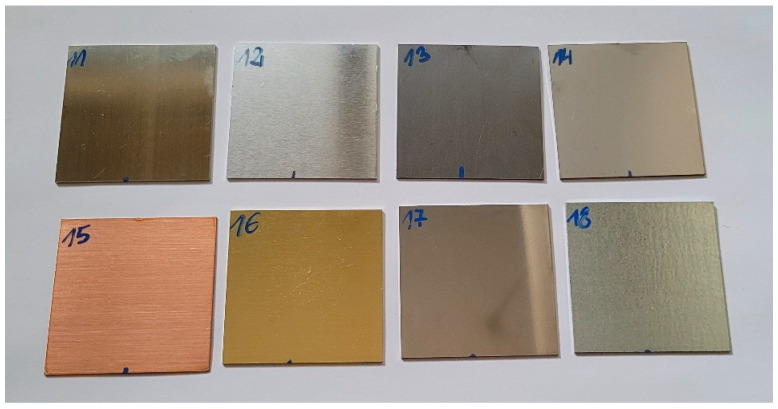
Samples for aging tests—metal samples only, numbered as described in Table 1.

Eighteen samples made from various materials were designated for testing. Table 1 provides the labels and descriptions of the samples, while Figure 1 and Figure 2 present images of the samples. Humidity during the entire test was maintained at a constant level under 30%.

The aging tests were conducted in a SUNTEST XLS+ aging chamber from Atlas Material Testing Technology GmbH (Linsengericht, Germany), in accordance with the applicable ISO 4892-1:2016 standard—Part 1 and Part 2. The SUNTEST device monitors light in the range of 300–800 nm, so the dose was recalculated for the 295–800 nm range for indoor light, which is 455 W/m^2^. The aging process parameters were set according to these calculations, resulting in a radiation dose equivalent to a one-month period of 169.8 MJ/m^2^, corresponding to a device operating time of 104.3 h at a temperature of 65 °C. Accelerated aging testing allows the long-term durability of materials to be assessed in days or weeks instead of years. The tests simulate operating conditions, which allows for determining potential degradation mechanisms and predicting the service life of the material. Intensive testing of samples may reveal problems that would only appear after years of use, e.g., brittleness, discoloration, and loss of mechanical properties. Shorter testing times also mean lower costs associated with testing and storing samples. Many industries (e.g., automotive, construction, and electronics) require aging tests as part of certification and approval for use.

To determine the impact of UV radiation on the mechanical properties of the tested materials, Shore D hardness tests, gloss measurements using a Gloss Meter (Termoprecyzja Company, Wroclaw, Poland), and mass measurements were conducted. The tests were performed after each consecutive four-month aging period, up to a total of twelve months.

The hardness of the plastic samples was determined according to the PN-EN ISO 868:2005 standard. The Shore hardness measurement involves assessing the resistance of the test sample to the penetration of an indenter needle with a specific shape and size, positioned at the base of the measuring instrument.

Gloss measurements were conducted using a Gloss Meter (Termoprecyzja Company, Wroclaw, Poland). The gloss measurement involves illuminating the surface with a specialized light source at an angle and measuring the intensity of reflected radiation at the same angle. Measurements were taken at different angles. Typically, the process begins at a 60-degree angle to determine the optimal measurement angle. If the surface has a gloss level between 10 and 70 GU (Gloss Units), it is considered semi-gloss, and a 60-degree angle is appropriate. If the reading is below 10 GU, the surface is matte, and a more accurate measurement can be obtained at an 85-degree angle. For surfaces with very high gloss levels (>70 GU), the most accurate results are achieved at a 20-degree angle.

The tested samples exhibited a wide range of gloss levels; therefore, measurements were conducted at all three angles: 20, 60, and 85 degrees. Due to the surface warping of sample No. 7 after aging, gloss measurement on its surface was not possible.

The mass of the samples was measured using a Radwag WTC 3000 laboratory scale (Radwag Balances and Scales, Radom, Poland). After each consecutive four-month aging period in the chamber, the samples underwent visual inspection.

## 3. Results and Discussion

### 3.1. Results of Mechanical Strength Tests at −50 °C and 25 °C

**Figure 3:**
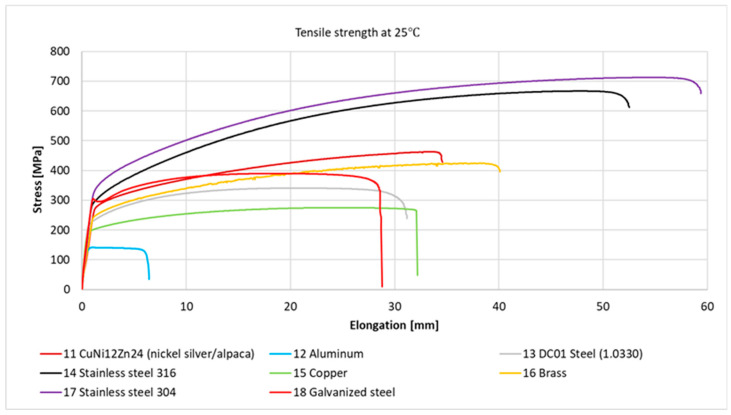
Graph of the strain–stress relationship for metal samples and their alloys under tensile stress at room temperature.

**Figure 4:**
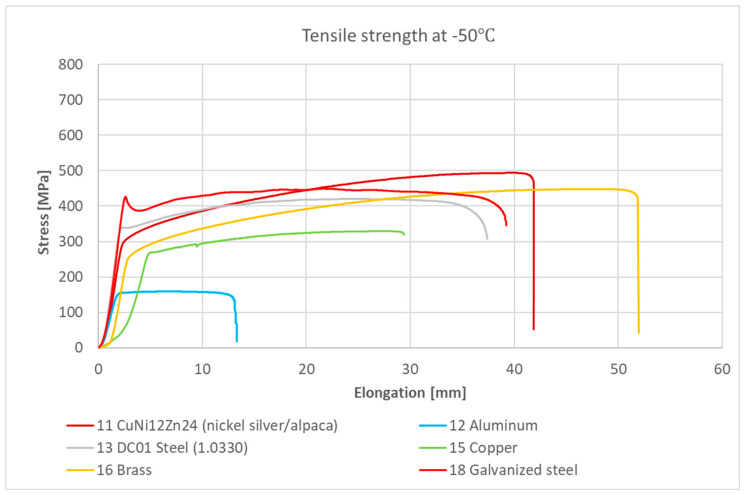
Graph of the strain–stress relationship for metal samples and their alloys under tensile stress at −50 °C.

Figure 3 and Figure 4 present the stress–strain relationships for individual samples of metals and their alloys. Representative curves were selected within each series based on the highest observed stress value.

The number of samples subjected to tensile testing was reduced from 8 to 6 materials due to their shape and surface morphology, as the engraving laminate and KRION mineral composite milling cutter did not meet the conditions required for the tensile test.

All metal samples and their alloys, except for sample No. 18—galvanized steel, exhibited strain–stress curves characteristic of ductile materials without a distinct yield point under room temperature conditions. For No. 18—galvanized steel, an elastic deformation region could be observed up to a stress level—depending on the sample—of approximately 284 to 308 MPa (at around 1% strain). Beyond this point, there was a slight increase in stress, followed by a decrease due to material yielding (within a strain range of approximately 1 to 2.5%). Subsequently, stress increased again with visible strain-hardening curves, indicating plastic deformation of the material.

**Figure 5:**
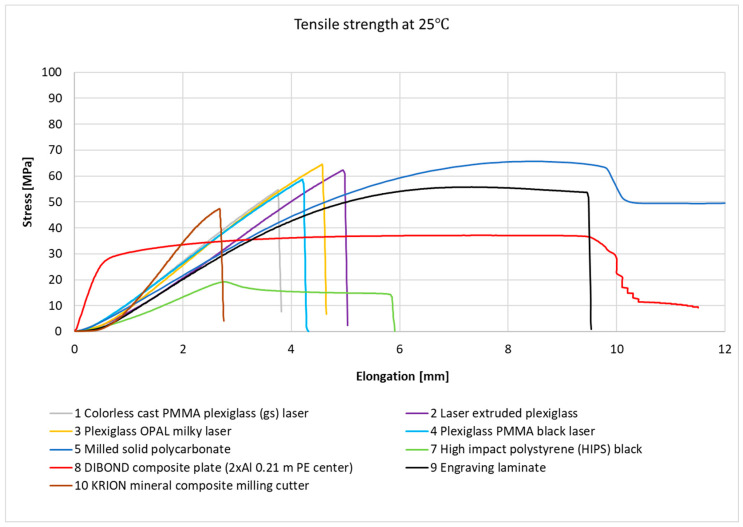
Graph of the strain–stress relationship for plastic samples under tensile stress at room temperature.

**Figure 6:**
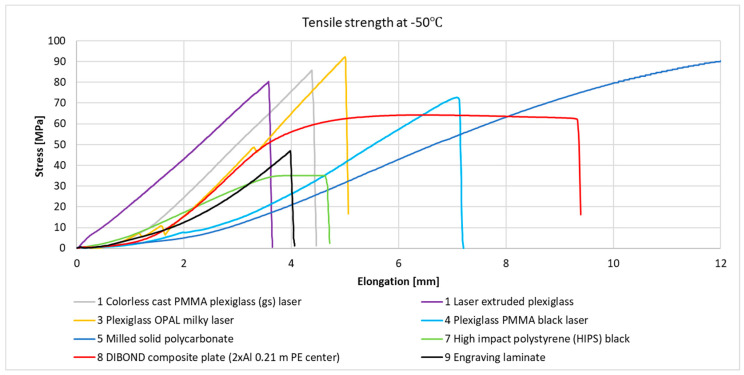
Graph of the strain–stress relationship for plastic samples under tensile stress at −50 °C.

Figure 5 and Figure 6 present the strain–stress relationships for individual plastic samples. Representative curves were selected within each series based on the highest observed stress value.

At room temperature, samples 5—solid polycarbonate, 6—milled solid polycarbonate, 8—DIBOND composite plate (2xAl 0.21 mm PE core), and 9—engraving laminate exhibited strain–stress curves typical of hard and durable plastics, with high elongation at break (138.80%, 12.26%, and 11.08%, respectively). However, at −50 °C, a significant decrease in elongation at break was observed for No. 6—milled solid polycarbonate (down to 35.42%) and for No. 9—engraving laminate (down to 5.36%). For No. 8—DIBOND composite plate (2xAl 0.21 mm PE core), a slight increase in elongation at break was observed (up to 12.98%), which fell within the standard deviation range for the mean value. This can be interpreted as no significant effect of temperature on the elongation at break for this sample. It is also important to note that No. 8—DIBOND composite plate (2xAl 0.21 mm PE core), is a hybrid material consisting of aluminum cladding and a polyethylene core, which also influences its behavior in mechanical tests.

All plexiglass samples and No. 10—KRION mineral composite milling cutter, displayed strain–stress curves characteristic of brittle materials at room temperature. At −50 °C, the plexiglass samples exhibited curves similar to those at room temperature, with differences mainly in elongation at break and maximum stress values. A significant change in curve behavior was observed for sample No. 7—high-impact polystyrene (HIPS) black. At room temperature, this material displayed curves typical of soft plastics, whereas at −50 °C, the curves resembled those of brittle materials.

### 3.2. Results of Aging Tests in the UV Chamber

**Figure 7:**
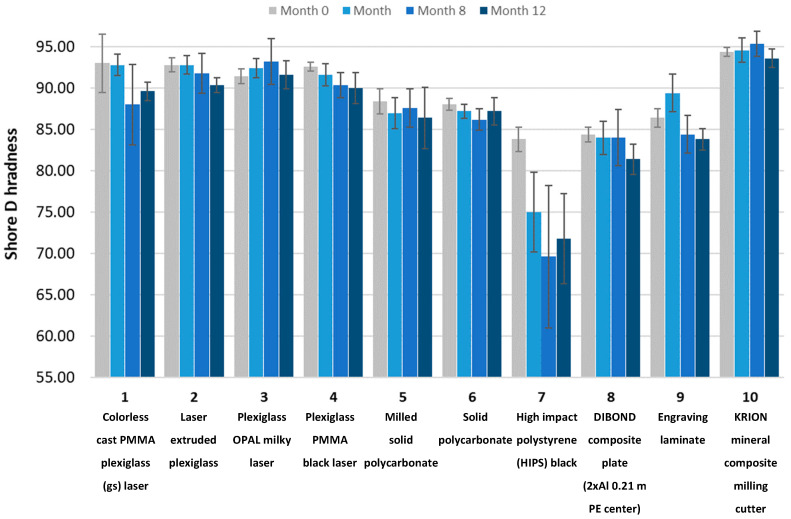
Shore D hardness of the tested plastic samples.

The initial analysis focused on the impact of degrading factors on the hardness of the samples. Figure 7 presents the hardness measurement results before the aging process and after 4, 8, and 12 simulated months of aging for the plastic samples.

Sample No. 7, high-impact polystyrene (HIPS), exhibited the largest decrease in hardness up to the 8-month aging mark. However, due to deformation after aging, the variation in results for this sample was substantial. For samples No. 3 and No. 10, a stable hardness was observed up to the 8th month of aging, followed by a decrease after 12 months.

Figure 7 shows an increase in the hardness of PMMA and polycarbonate after prolonged aging, which can be attributed to UV-induced surface changes. UV radiation promotes oxidative degradation, polymer chain scission, and surface embrittlement, all of which contribute to increased hardness. Studies have shown that polycarbonate hardness can rise significantly after UV exposure, even within the first hour, while FTIR-detectable structural changes appear later [31]. This process leads to reduced molecular weight and surface erosion, resulting in stiffer yet more brittle materials—explaining the observed hardness increase despite the overall mechanical deterioration.

**Figure 8:**
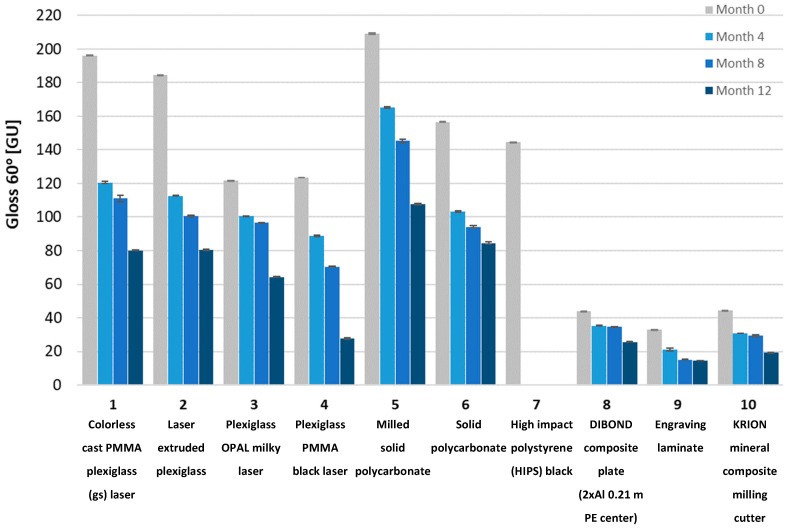
Gloss measured at a 60° angle for plastic samples.

**Figure 9:**
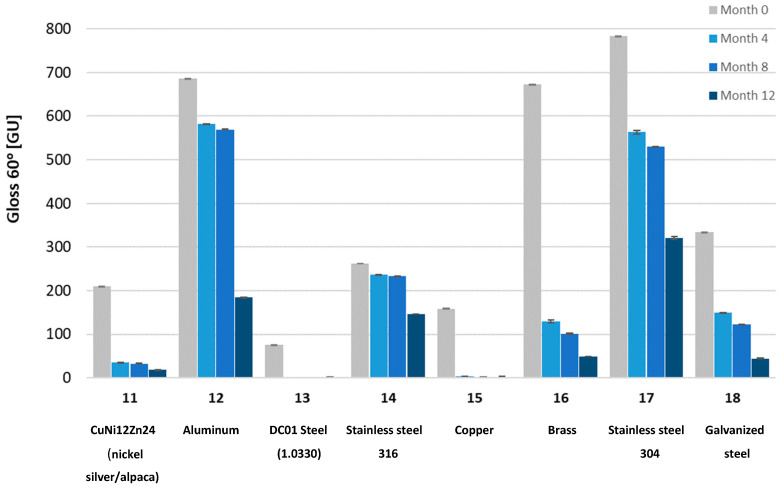
Gloss measured at a 60° angle for metal samples.

**Figure 10:**
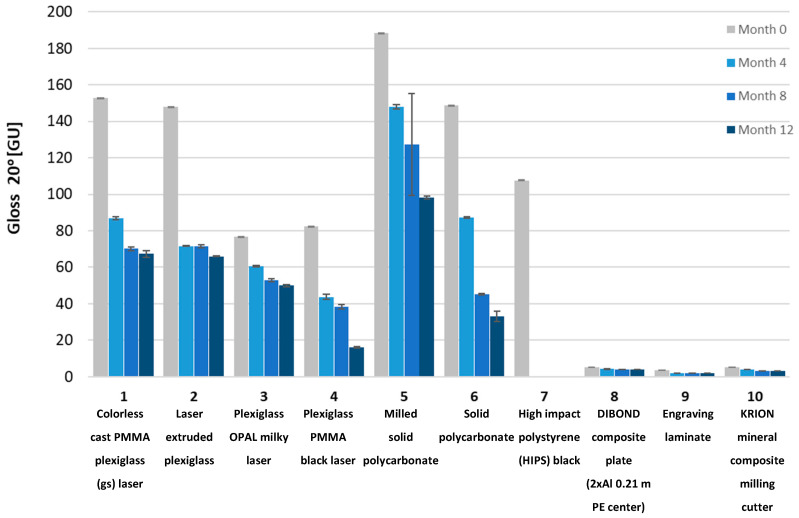
Gloss measured at a 20° angle for plastic samples.

**Figure 11:**
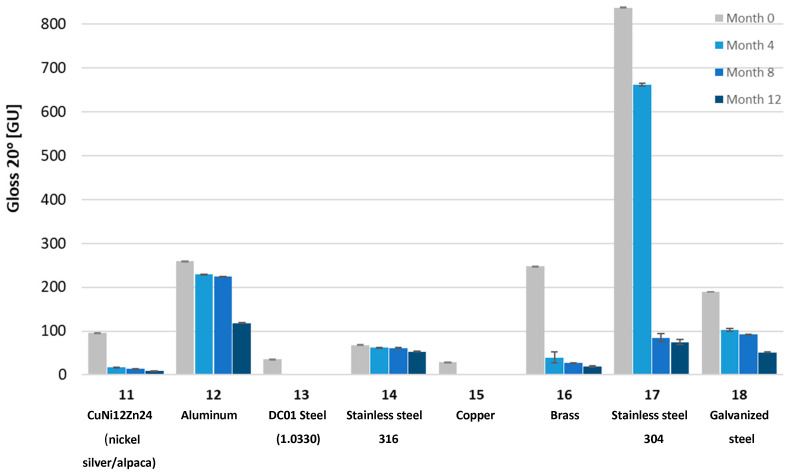
Gloss measured at a 20° angle for metal samples.

**Figure 12:**
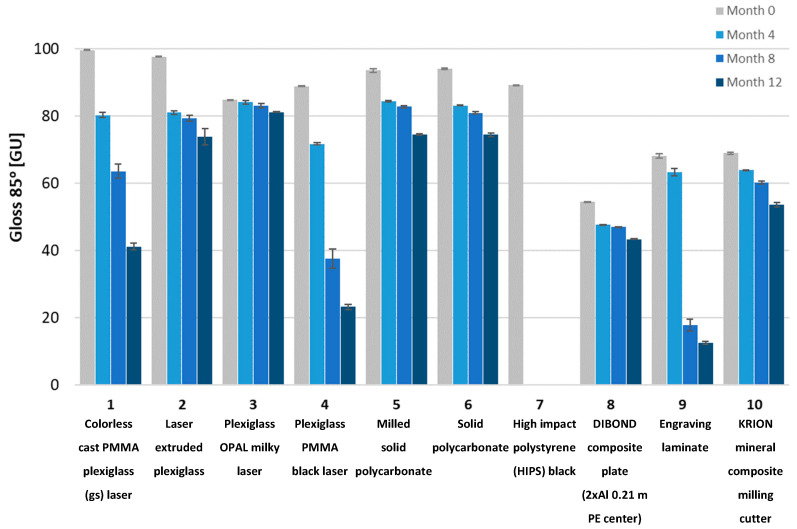
Gloss measured at an 85° angle for plastic samples.

**Figure 13:**
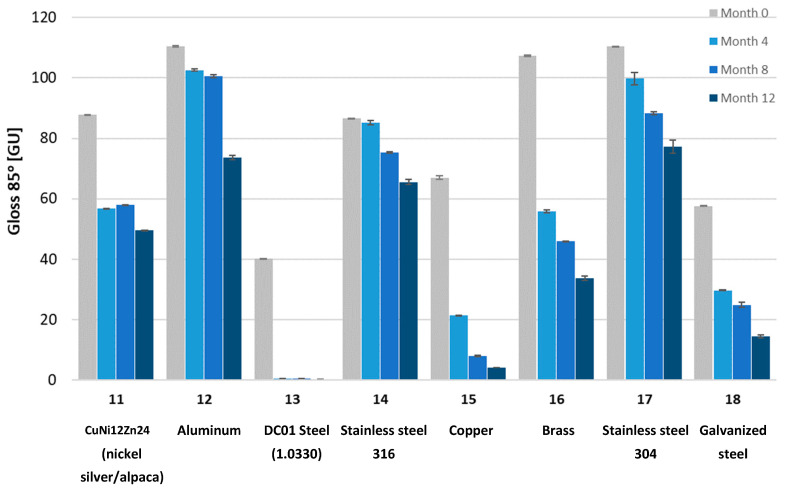
Gloss measured at an 85° angle for metal samples.

Gloss measurements were then conducted. The tested samples exhibited a wide range of gloss levels, so measurements were taken at all three angles: 20, 60, and 85 degrees. Due to surface warping of sample No. 7 after aging, the gloss measurement on its surface was not possible. The gloss measurement results for all samples at the three angles are shown in Figure 8, Figure 9, Figure 10, Figure 11, Figure 12 and Figure 13. The numbers on the charts correspond to the sample numbering provided in Table 2.

The loss of gloss of materials after the aging process is a phenomenon resulting from a number of physical and chemical processes occurring in the material. Under the influence of UV light, the polymer oxidizes, which causes microcracks and a change in the surface structure. Then, the ability to reflect light decreases, and thus the gloss decreases. Under the influence of moisture, among others, in polycarbonates, hydrolysis occurs, which increases the surface roughness. The influence of temperature is also not a neutral factor in the loss of gloss. As a result of heating and cooling, thermal stresses occur, which cause microcracks and surface dulling. On the copper surface (sample No. 15), the phenomenon of oxidation occurred, as a result of which black copper (II) oxide was formed. Also, in sample No. 16 (brass), there was a significant decrease in gloss. The optical properties of the surface changed under the influence of temperature and moisture [32].

The next stage of the study involved measuring the mass of the samples using a Radwag WTC 3000 laboratory scale. Table 2 summarizes the mass values for all samples. The mass of the samples showed minimal changes, with weight fluctuations not exceeding ±0.09 g. For most samples, the mass remained stable throughout the entire aging period.

The mass measurement of the samples did not show significant changes. Therefore, it can be assumed that the changes occurred mainly in the structure and properties without a noticeable impact on the sample mass.

After each consecutive four-month aging period in the chamber, the samples underwent visual inspection. Sample No. 7, made of high-impact polystyrene, developed warping. This occurred due to the elevated temperature in the chamber and UV radiation, with the effect likely intensified by the black color of the sample. Some plastic samples also showed discoloration in the form of yellow spots.

**Figure 14:**
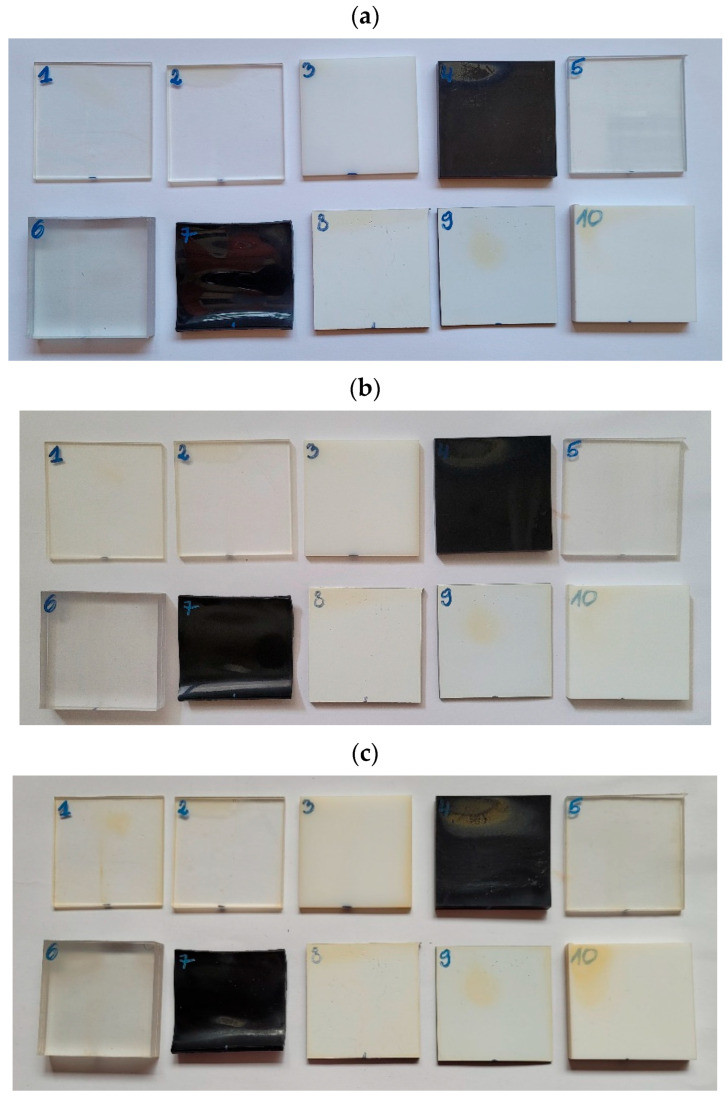
Plastic samples (**a**) after 4 months, (**b**) after 8 months of aging, and (**c**) after 12 months of aging (the numbers written on the samples refer to Table 2).

**Figure 15:**
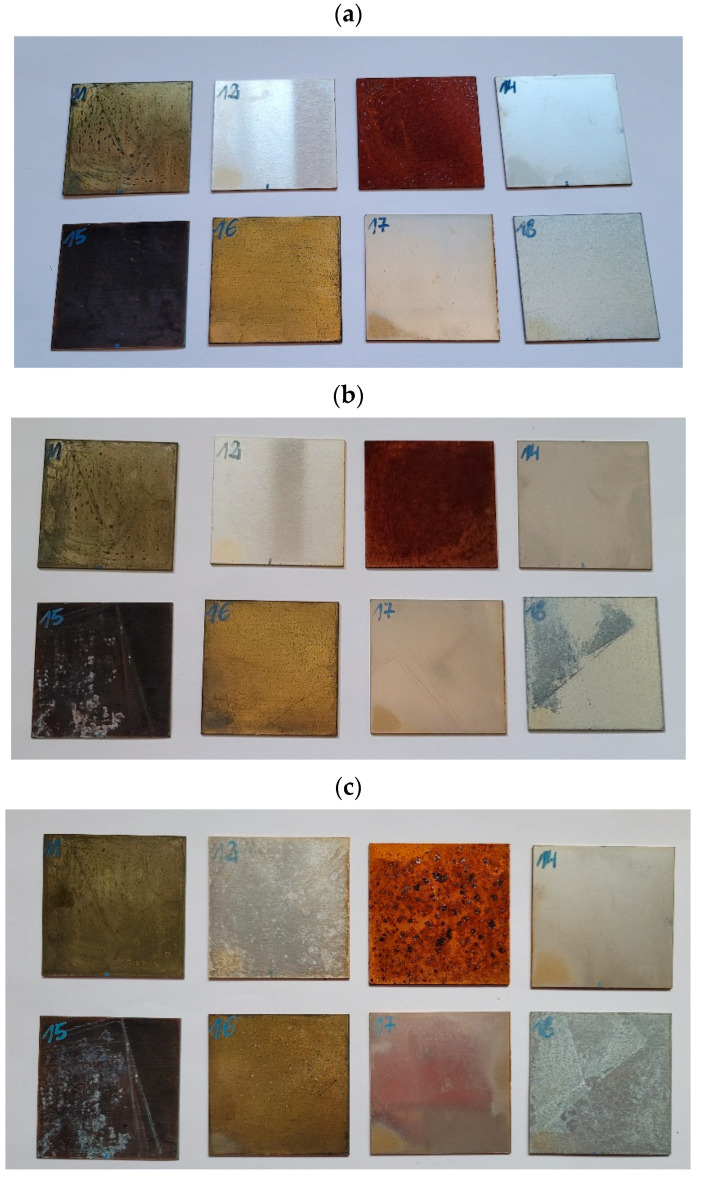
Metal samples (**a**) after 4 months, (**b**) after 8 months of aging, and (**c**) after 12 months of aging (the numbers written on the samples refer to Table 2).

For the metal samples, corrosion developed across the entire surface of DC01 steel (sample No. 13). The least visual changes were observed in samples No. 12, No. 14, and No. 17. Photos of the plastic and metal samples are shown in Figure 14 and Figure 15.

**Figure 16:**
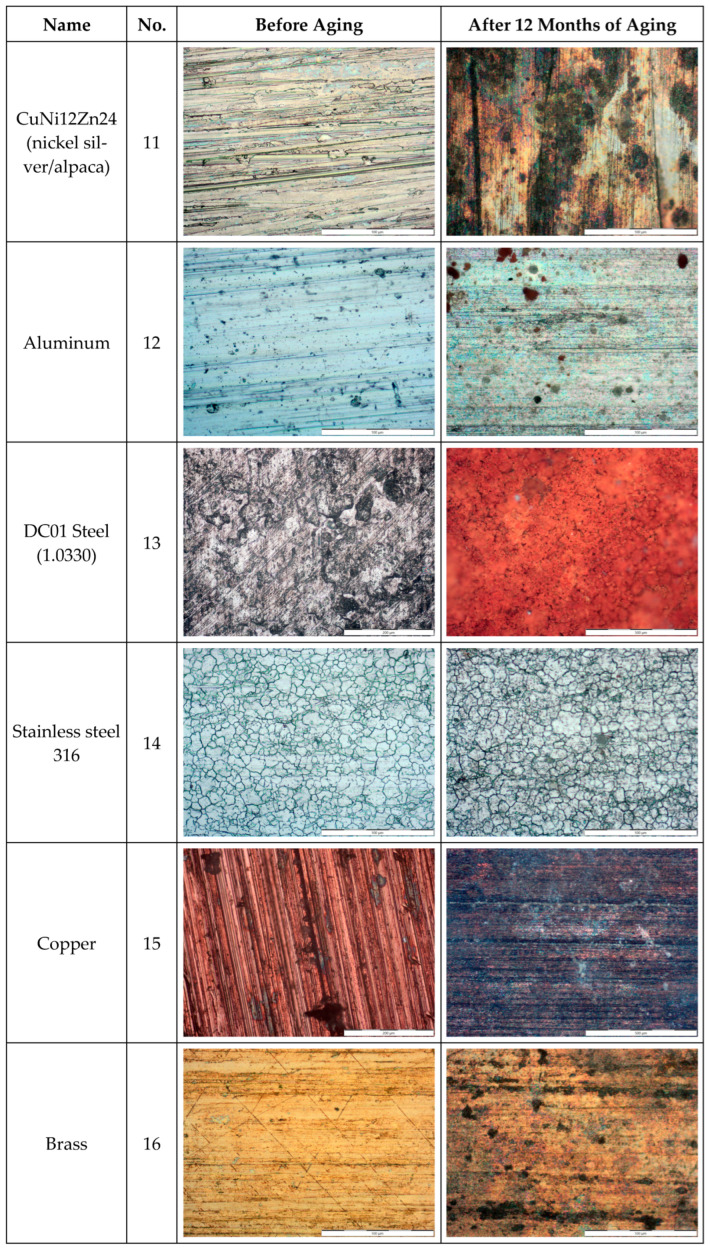

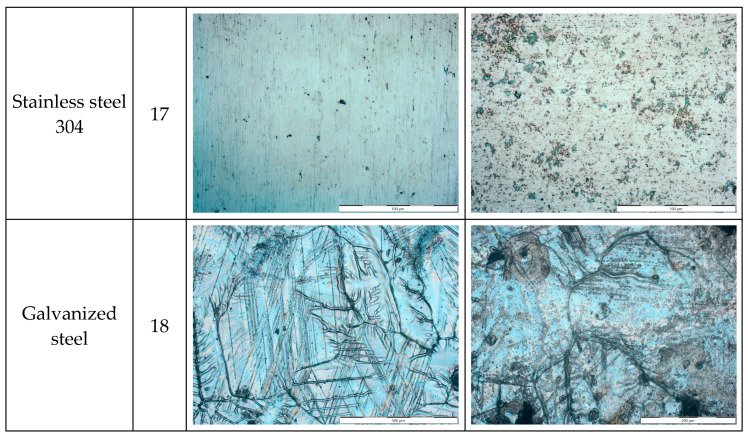
Microscopic view of metal samples before aging and after 12 months of artificial aging.

The metal samples were additionally analyzed to assess the changes that occurred on their surfaces. These changes were observed using an Olympus GX51 optical microscope at magnifications of up to 1000× and presented in Figure 16.

The microscopic examination of metal samples after 12 months of artificial aging revealed significant differences in their resistance to environmental factors typical for temperate climate conditions. The most dramatic transformation was observed in sample No. 13 (DC01 steel), which exhibited extensive corrosion across its entire surface, transitioning from a grayish metallic appearance to a uniform reddish-brown color characteristic of iron oxide formation. This rapid deterioration began within days of exposure to the aging conditions, with complete loss of surface gloss after the 12-month period. Sample No. 15 (copper) demonstrated a substantial color shift from its initial bright reddish tone to a dark brown-graphite appearance, representing the progressive formation of copper oxide layers, consistent with natural patination processes. Similarly, sample No. 16 (brass) showed considerable tarnishing, with scattered dark spots appearing on its previously uniform golden surface, likely due to preferential oxidation of zinc in the copper-zinc alloy. The CuNi12Zn24 sample (No. 11) displayed significant surface degradation with visible corrosion products and loss of its original silver-like appearance. In stark contrast, samples No. 12 (aluminum), No. 14 (stainless steel 316), and No. 17 (stainless steel 304) maintained their surface integrity with minimal visual changes, demonstrating the protective nature of their native oxide films. Sample No. 14 particularly retained its characteristic crystalline structure visible in the microscopic view, with only slight dulling of its surface. These observations, particularly for aluminum and stainless steels, showed the smallest decrease in reflective properties, confirming their superior resistance to degradation under prolonged exposure to destructive environmental factors, including UV radiation, temperature fluctuations, and humidity [33].

### 3.3. Discussion

The conducted research revealed significant differences in the behavior of metallic and polymeric materials under the destructive influence of environmental factors characteristic for a temperate climate. The obtained strength results indicated that low temperature (−50 °C) had a particularly significant effect on the mechanical properties of plastics, which confirmed previous observations regarding changes in the behavior of polymers at low temperatures, as described by Callister and Rethwisch [34]. The drastic change in the character of the stress–strain curves for sample No. 7 (high-impact polystyrene, HIPS), which at room temperature showed features of a soft plastic and at −50 °C behaved like a brittle material, was consistent with the fundamental principles of materials science concerning the glass transition of polymers. Aging tests confirmed that long-term exposure to UV radiation and variable humidity leads to significant changes in surface properties, such as gloss and hardness, which corresponds to the degradation mechanisms of materials described in the “*Handbook of Material Weathering*” [35].

Particularly noteworthy is the high resistance to atmospheric factors demonstrated by aluminum and stainless steels 304 and 316 among metals, and by laser-extruded PMMA, milky OPAL PMMA, and polycarbonate among plastics. According to the data contained in the [36], these properties can be attributed to surface passivation in stainless steels and the stability of the aluminum oxide layer. Simultaneous testing of these materials at low temperatures and under accelerated aging allowed for a comprehensive assessment of their suitability for use in outdoor applications in a temperate climate.

From a practical point of view, the results indicate that when designing structural elements, housings, or covers operating outdoors in a temperate climate, it is crucial to consider both long-term resistance to atmospheric factors and the behavior of the material at extreme temperatures. This is especially important for plastics, whose properties may change drastically at sub-zero temperatures, potentially leading to unexpected failures during winter operation. Corrosion test results for DC01 steel (sample No. 13) showed that for non-alloy steels, it is necessary to use appropriate protective coatings, which confirms the classic corrosion mechanisms described in the literature.

In summary, the research provided valuable information on the behavior of various materials in temperate climate conditions, indicating aluminum, brass, and stainless steels 304 and 316 among metals, and laser-extruded PMMA, milky OPAL PMMA, and polycarbonate among plastics, as materials with the highest resistance to destructive environmental factors. These results are directly applicable to engineering practice, enabling a more informed selection of materials for specific outdoor applications, which can increase the durability and reliability of products and reduce maintenance and operating costs.

## 4. Summary and Conclusions

During the aging process, the hardness of the samples underwent changes, with a decrease in hardness observed for most of the plastic samples, amounting to a few percent. Sample No. 10 (KRION) exhibited the highest hardness (approximately 90 ShD). Sample No. 7 (HIPS) showed the greatest decrease in hardness after eight months of aging. The measurement results for this sample were highly divergent, which also resulted from the sample deformation as early as after four months of aging.

The mass of the samples underwent minimal changes, with fluctuations in weight not exceeding ± 0.09 g. For most samples, the mass remained at the same level throughout the aging period. Aging (temperature increase and rainfall) did not result in weight gain, indicating that the samples are not hygroscopic. Moreover, no reduction in sample mass was observed, which may accompany the degradation processes of polymeric materials.

The aging process drastically affected the gloss change of all samples. Sample No. 13 (DC01 steel) completely lost its gloss after 12 months of aging. The smallest change in gloss occurred for samples No. 12 (aluminum), No. 14 (acid-resistant), and No. 17 (stainless steel). Samples No. 1 and No. 2 of PMMA (polymethyl methacrylate) transparent and No. 5 of PC (polycarbonate) lost approximately 50% of their gloss (measured at a 60° angle). In sample No. 4 (black acrylic), the gloss decreased tenfold after 12 months of aging. Sample No. 7, made of high-impact polystyrene, deformed after four months of aging, wrinkling, which prevented gloss measurements.

Deep observations led to the conclusion that sample No. 3 (opaque milk PMMA) was yellowed. Yellow discolorations appeared also on the surface of samples No. 8 (DIBOND), No. 9 (engraving laminate), and No. 10 (KRION). Samples No. 5 (polycarbonate) and No. 6 (milled polycarbonate) exhibited the smallest visual changes after the aging process. DC01 steel (sample No. 13) corroded within a few days of the aging process initiation in the chamber. Copper (sample No. 15) changed color from copper to dark brown to graphite after four months of aging. The smallest visual changes occurred in samples No. 14 (acid-resistant) and No. 17 (stainless steel).

The conducted research led to the following conclusions:

- In a temperate climate, where materials are exposed to low and high temperatures as well as variable humidity, it is essential to test the material’s durability if it is to serve and operate as a housing or protective cover.
- Selected plastic materials (laser-extruded plexiglass, milky laser plexiglass OPAL, milled solid polycarbonate, and solid polycarbonate) provided satisfactory resistance to atmospheric factors with minimal hardness loss measured by the Shore method.
- Selected metals (aluminum and stainless steel 304) also demonstrated high resistance to atmospheric factors in terms of appearance after 12 months of aging
- For specific applications, it is advisable to anticipate the behavior of materials at reduced temperatures down to −50 °C, as this may be crucial for plastics, some of which (e.g., plexiglass) experience a strength change of over 30%.
- Among metals and their alloys, aluminum, brass, and stainless steel 304 and 316 can be used, and among plastics, laser-extruded plexiglass, milky laser plexiglass OPAL, milled solid polycarbonate, and solid polycarbonate are recommended as materials with sufficient resistance to destructive factors in the temperate climate zone.

## Figures and Tables

**Table 1:** List of samples used for aging tests and for tensile strength tests, including test parameters and conditions.

			Test Conditions			Properties of Material Samples Intended for Testing
			Temperature 25 °C	Temperature −50 °C		
No.	Material	Thickness (mm)	Measurement Speed (mm/min)	Measurement Speed (mm/min)	Conditioning Time (min)	
1	Colorless cast PMMA plexiglass (gs) laser	3	50	50	10	Purchased as new, with factory-applied protective film; the film was removed immediately prior to submission for testing.
2	Laser-extruded plexiglass	3	50	50	10	
3	Plexiglass OPAL milky laser	3	50	50	10	
4	Plexiglass PMMA black laser	5	50	50	10	
5 & 6	Milled solid polycarbonate	3	50	50	10	
7	High-impact polystyrene (HIPS) black	2	50	50	10	
8	DIBOND composite plate (2xAl 0.21 m PE center)	3	50	50	10	
9	Engraving laminate	2	50	50	10	
10	KRION mineral composite milling cutter	5	50	50	-	Purchased as new, supplier: ANTALIS POLSKA. Side A was factory-sealed with a protective film.
11	CuNi12Zn24 (nickel silver/alpaca)	1	9	9	5	Purchased as new, in the form of metal sheets, without protective film, exposed to atmospheric conditions, but polished with 1000-grit sandpaper to refresh the surface before being submitted for testing.
12	Aluminum	1	9	9	5	Purchased as new, in the form of aluminum sheet panels without protective film, sourced from the supplier’s warehouse. Some exposure to atmospheric conditions was inevitable, but the surface remained fresh.
13	DC01 Steel (1.0330)	1	9	9	5	Purchased as new, without protective film, factory-delivered without signs of corrosion.
14	Stainless steel 316	1	9	9	-	Purchased as new, in the form of metal sheet panels, with factory-applied protective film; the film was removed immediately before testing.
15	Copper	0,5	9	9	5	Purchased as new, in the form of metal sheet panels, without protective film, exposed to atmospheric conditions, but brushed with 1000-grit sandpaper to refresh the surface before submission for testing.
16	Brass	1	9	9	5	Purchased as new, in the form of metal sheet panels, without protective film, exposed to atmospheric conditions, but brushed with 1000-grit sandpaper to refresh the surface before being submitted for testing.
17	Stainless steel 304	1	9	9	-	Purchased as new, in the form of metal sheet panels, with factory-applied protective film; the film was removed immediately before the start of testing.
18	Galvanized steel	1	9	9	5	Purchased as new, without protective film, factory-delivered on a pallet without signs of corrosion, as it has a galvanic coating. There was exposure to atmospheric conditions, but it did not affect the coating.

**Table 2:** Results of sample weight measurements.

No. of the Sample	Material	Average Weight (g)			
		Month 0	Month 4	Month 8	Month 12
1	Colorless cast PMMA plexiglass (gs) laser	5.58	5.6	5.56	5.62
2	Laser-extruded plexiglass	8.95	8.97	8.97	9
3	Plexiglass OPAL milky laser	8.9	8.92	8.92	8.94
4	Plexiglass PMMA black laser	14.41	14.44	14.42	14.42
5	Milled solid polycarbonate	8.37	8.39	8.36	8.34
6	Solid polycarbonate	28.97	29.05	29.01	29.04
7	High-impact polystyrene (HIPS) black	5.01	5.02	5.01	5
8	DIBOND composite plate (2xAl 0.21 m PE center)	8.75	8.73	8.66	8.7
9	Engraving laminate	4.37	4.34	4.33	4.33
10	KRION mineral composite milling cutter	26.13	26.15	26.22	26.27
11	CuNi12Zn24 (nickel silver/alpaca)	21.38	21.34	21.32	21.43
12	Aluminum	6.61	6.62	6.61	6.61
13	DC01 Steel (1.0330)	19.65	19.53	19.56	19.79
14	Stainless steel 316	19.32	19.33	19.34	19.33
15	Copper	11.09	11.08	11.08	11.07
16	Brass	21.49	21.49	21.45	21.42
17	Stainless steel 304	19.32	19.33	19.32	19.32
18	Galvanized steel	19.44	19.38	19.28	19.3

## Data Availability

The datasets presented in this article are not readily available because the data are part of an ongoing study or due to technical limitations. Requests to access the datasets should be directed to m.kogut@metaloplastyka.eu.
